# Strive to Reduce Slurry Erosion and Cavitation in Pumps through Flow Modifications, Design Optimization and Some Other Techniques: Long Term Impact on Process Industry

**DOI:** 10.3390/ma14030521

**Published:** 2021-01-21

**Authors:** Adnan Aslam Noon, Absaar Ul Jabbar, Hasan Koten, Man-Hoe Kim, Hafiz Waqar Ahmed, Umair Mueed, Ahmad Adnan Shoukat, Bilal Anwar

**Affiliations:** 1 Department of Mechanical Engineering, FET. International Islamic University, Islamabad 44000, Pakistan; adnan.aslam@iiu.edu.pk (A.A.N.); bilal.bsme353@iiu.edu.pk (B.A.); 2 Research Centre for Modelling and Simulation, National University of Sciences and Technology (NUST), Islamabad 44000, Pakistan; absaar@rcms.nust.edu.pk; 3 Department of Mechanical Engineering, Istanbul Medeniyet University, İstanbul 34700, Turkey; hasan.koten@medeniyet.edu.tr; 4 School of Mechanical Engineering & IEDT, Kyungpook National University, Daegu 41566, Korea; 5 Department of Mechanical Engineering, Sungkyunkwan University, Natural Sciences Campus: (16419), Seobu-ro, Jangan-gu, Suwon-si, Gyeonggi-do 2066, Korea; waqar543@skku.edu; 6 Department of Mechanical Engineering, University of Engineering and Technology, Taxila 47050, Pakistan; umair.mueed@gmail.com; 7 Department of Mechanical Engineering, Institute of Space Technology, Islamabad 44000, Pakistan; ahmad.adnan@ist.edu.pk

**Keywords:** centrifugal pump, erosion, cavitation, CFD, process industry

## Abstract

Centrifugal pumps are being widely used in various industries for moving fluids that carry solids through pipelines where the need of head and flow rate is not high. Slurry erosion and cavitation are an extremely complex and not yet fully understood phenomenon that occur in centrifugal pumps; however, these undesirable phenomena can be reduced to a certain extent. Appropriate design and development of experiments is required to reasonably predict slurry erosion and cavitation. However, CFD methodology complements analytical solutions and experiments whenever testing of equipment has limitations. The current paper highlights the various slurry erosion and cavitation reduction techniques utilized by different researchers. Economic analysis conducted for a case study relevant to centrifugal pump (CP) usage in Pakistan shows that an 8% enhancement in pump efficiency can reduce the life cycle cost to about 17.6%, which could save up to USD 4281 for a single pump annually in Pakistan.

## 1. Introduction

Pumps are the most important industrial equipment used for the transportation of liquids carrying solids. A centrifugal pump (CP) is commonly used in numerous industries for this purpose for small and medium distance applications. The dredging industry involves the largest quantities of liquid–solid mixture throughputs obtained through the application of very large CPs. The mining industry uses CPs to transport large amounts of ore concentrates (such as coal, precious minerals and iron, etc.) mixed with water to the destination. Pulp slurry in the paper industry is moved to various papermaking stages with the help of centrifugal pumps. Similarly, petrochemical, food processing and pharmaceutical industries are amongst many other industries that employ the use of slurry CPs. This transport equipment is, therefore, at the heart of many industries and its proper performance is vital for the smooth operations of these industries.

Slurry erosion and cavitation are the two most common causes that highly contribute to the performance deterioration and reduced lifespan of centrifugal pumps. Slurry erosion occurs when the solid particles entrained in the liquid flow impact the pump impeller or volute casing with high velocity. Cavitation arises due to the pressure fluctuations in pumps, resulting in vapor bubbles forming in low-pressure zones which implode when reaching the high-pressure zones. Both slurry erosion and cavitation cause damage to metal surfaces affecting the shape of impeller blades and volute casing. The damaged surfaces alter the flow characteristics significantly which play vital role in furthering the erosion process [1]. This phenomenon results in the loss of hydraulic performance of the pump on one hand, and increased noise and vibrations on the other hand. The induced vibrations are often so severe that they can cause damage to the pump parts (bearings, mechanical seals, shafts, etc.) and even to the pump’s foundation and piping system as well. Replacement of the defective parts not only adds to the maintenance costs, but also increased breakdown times and production losses.

To mitigate the effects of slurry erosion and cavitation as mentioned above, it is critical to better understand, predict and reduce these phenomena. Regarding the CP performance, several researchers have worked on the slurry erosion in centrifugal pumps through experiments and simulations. Active areas of research and development, characteristics of settling or non-settling slurries, operational and geometrical parameters such as pump flow rate, pump rotational speed, particle diameter, tongue curvature, slope of the discharge pipe and casing width are all identified and discussed [2,3,4,5].

The current review summarizes methods for the prediction of slurry particle erosion and cavitation detection and reduction in various types of pumps. It has been identified that experimental work completed so far lacks similarity with the field studies. A realistic and cost-effective solution is necessary in order to enhance the pumping performance. Gaps in the literature are also identified; FSI, coalesced effects, surface roughness, etc. are rarely discussed by the researchers. Furthermore, economic analysis has been performed by utilizing a case study relevant to CP usage in Pakistan. It has been shown that an 8% enhancement in pump efficiency can reduce the life cycle cost to about 17.6% which could save USD 4281 for a single pump annually.

## 2. Significance of Slurry Erosion and Cavitation

To maintain uniform production levels in a process industry, proper performance of the transport equipment is imperative. Liquid-solid mixtures cause erosion in industrial equipment e.g., in pipeline parts, pumps, heat exchangers and in several other applications which results in equipment damage, efficiency drops and failure.

### 2.1. Slurry Erosion

**Figure 1:**
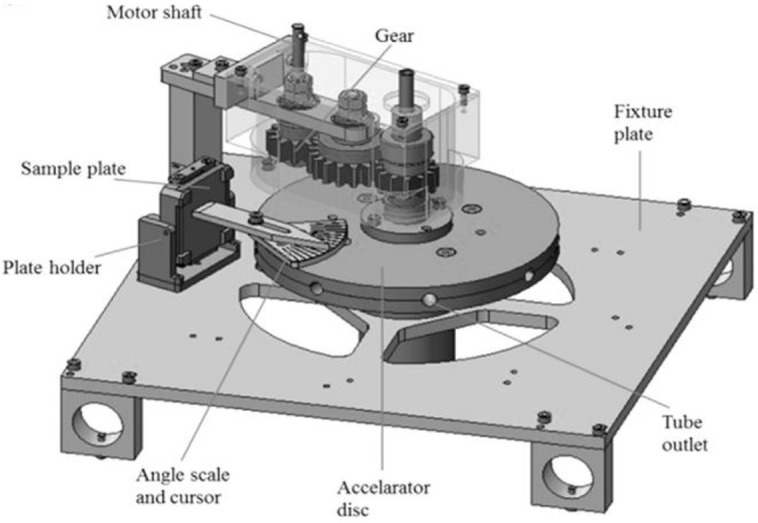
Centrifugal accelerator erosion tester model comprising of eight internal tubes [7].

Slurry erosion occurs when liquid containing solid particles interacts with a target surface which experiences a loss of material. Solid particles may vary in diameter, shape and concentration depending on the nature of slurry flow. Slurry could be either settling or non-settling which depends on the flow dynamics of the liquid. Many people have worked on the significance of erosion and cavitation in several different ways. Walker and Robbie [6] compared the erosion life of a particular material in the field with that in the laboratory erosion tests. The laboratory tests included different tests; i.e., erosion jet tester, commercial dry sand rubber wheel (DSRW) abrasion, slurry jet erosion (SJE) and Coriolis erosion. They explained that various erosion rates obtained between the laboratory and field tests were in contradiction. It happens due to lack of understanding of particular erosion conditions in the pump as well as samples microstructure. Azimian et al. [7] designed a centrifugal erosion tester to examine the particle motion. They have quantified the hydro abrasion of materials caused by the slurry flow. The flow simulation results were compared and validated through experimental data. Figure 1 shows the details of the apparatus which is similar to the erosion situations found usually in industry. In total, eight acceleration tubes are mounted on a disc, which is the unique part of the equipment.

**Figure 2:**
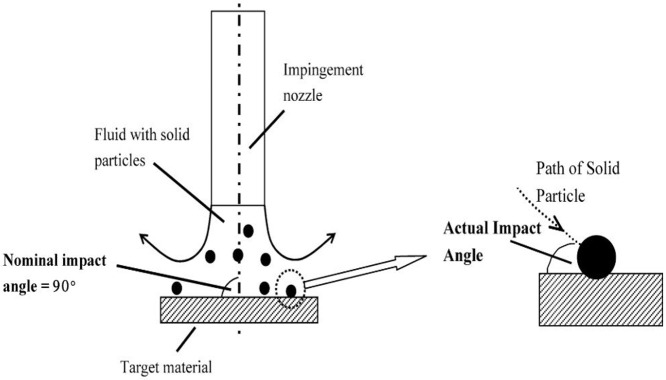
Actual and nominal impact angles are compared for solid particles [11].

Christopher et al. [8] emphasized the significance of CFD analysis to understand the erosion phenomena, the maximum erosion depth was measured to be approximately four times and the scar was predicted to have a typical V-shape which was not shown in the experimental data. Lin et al. [9] compared the erosion experimental results under different sand rates to show the effect of sand flow rate and its effect on particle velocities and erosion. It is also identified that particle shape influences erosion rate more than particle size for the presented conditions. Kruger et al. [10] demonstrated that particle impingement angle and solids concentration play a dominant role in shock-like processes as they occur along the leading edge of an impeller blade. Gnanavelu et al. [11] presented a novel technique for erosion–corrosion loss in various equipment which couples standard laboratory tests with numerical simulations to predict material wear rates through CFD techniques. Quantitative comparison is made between a metal sample in a standard 90° jet impingement test and for the improved geometry of an inclined sample. Figure 2 shows actual and assumed particle impact angles.

**Figure 3:**
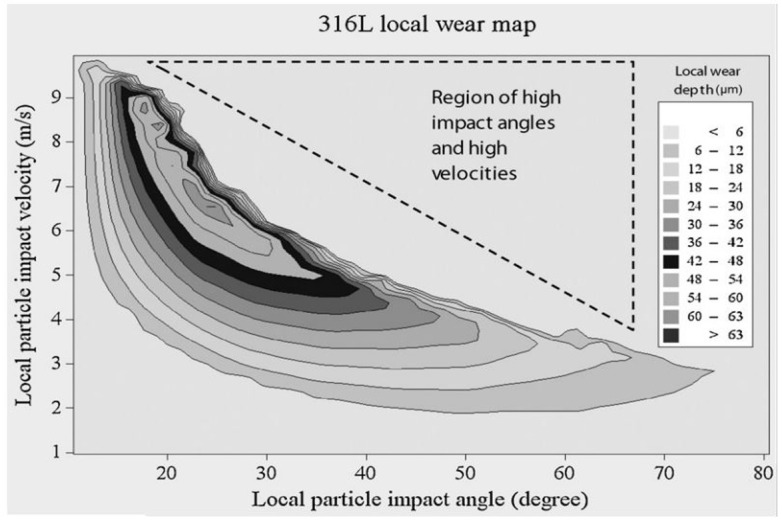
Wear data explained through the material-specific wear map for a duration of 120 min and for a variable jet velocity [11].

CFD simulations were utilized to correlate the local wear rates at different locations along the sample. A material-specific wear map is obtained from both testing and simulations as shown in Figure 3. The wear map can then be used to obtain material wear data for several particle impingement velocities with a maximum velocity of 10 m/s.

### 2.2. Cavitation

**Figure 4:**
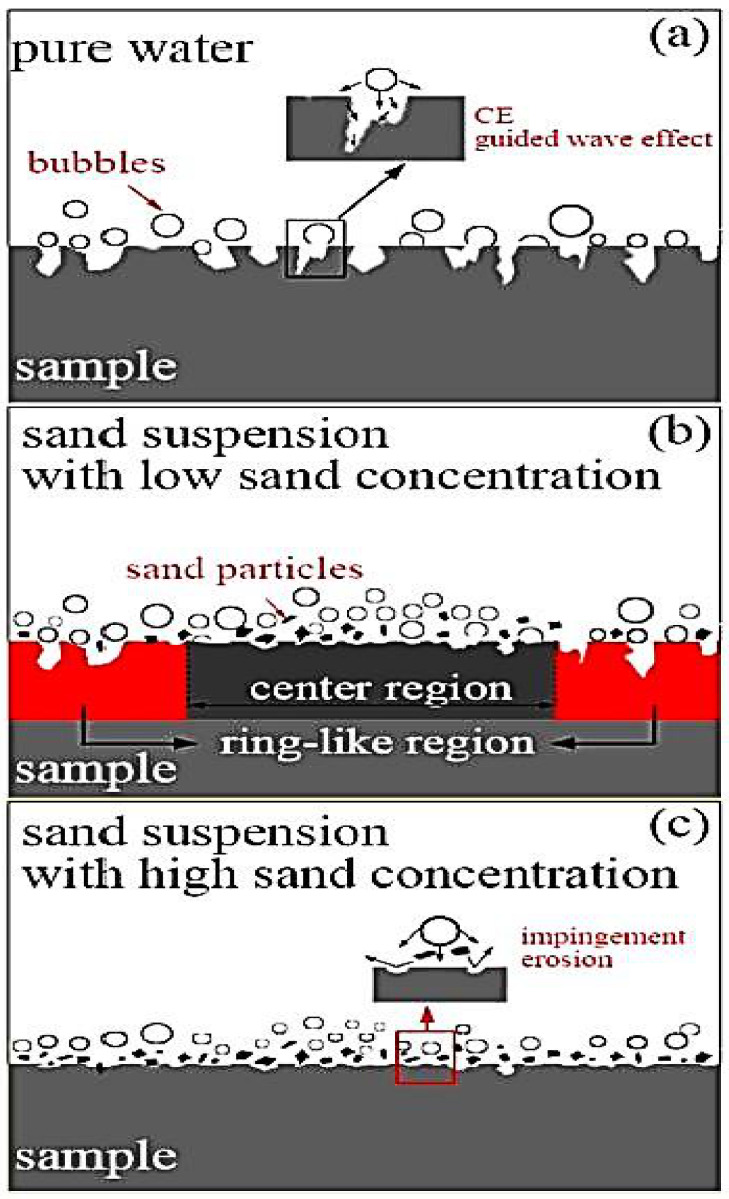
CE and CSE mechanisms (**a**–**c**) [13].

Cavitation phenomenon gives rise to many undesirable and unavoidable events such as implosion and explosion of bubbles, bubble bursts, generation of shock waves, etc. Experimental work for the cavitation detection and the efforts for its reduction have been occurring for a long time. The effect of cavitation is usually found at the suction side of the pump and at low pressure locations. Rao and Buckley [12] experimentally investigated the effect of cavitation on aluminum alloyed metal in three distinct liquids: distilled water, tap water and viscous mineral oil. They found the changes in roughness value and depth due to attack of cavitation. Furthermore, they showed the SEM images for the generation of pits, erosion and surface deformation. Hu et al. [13] compared experimentally the cavitation erosion(CE) in water and cavitation silt erosion(CSE) in suspended sand on a graded stainless steel. They evaluated the erosion damage through mass loss and scanning electron microscopy. The particles’ effect on sand concentrations on the CSE were considered as a significant parameter for cavitation erosion. The wear behavior can be seen as shown in Figure 4. For the cavitation erosion in pure water (Figure 4a), the material loss is due to the mechanisms of shock wave or micro jet flow. By adding sand particles with small concentration (Figure 4b), the center region is subject to impact erosion and the quantity of big CE holes decrease. Figure 4c shows the particle impingement.

**Figure 5:**
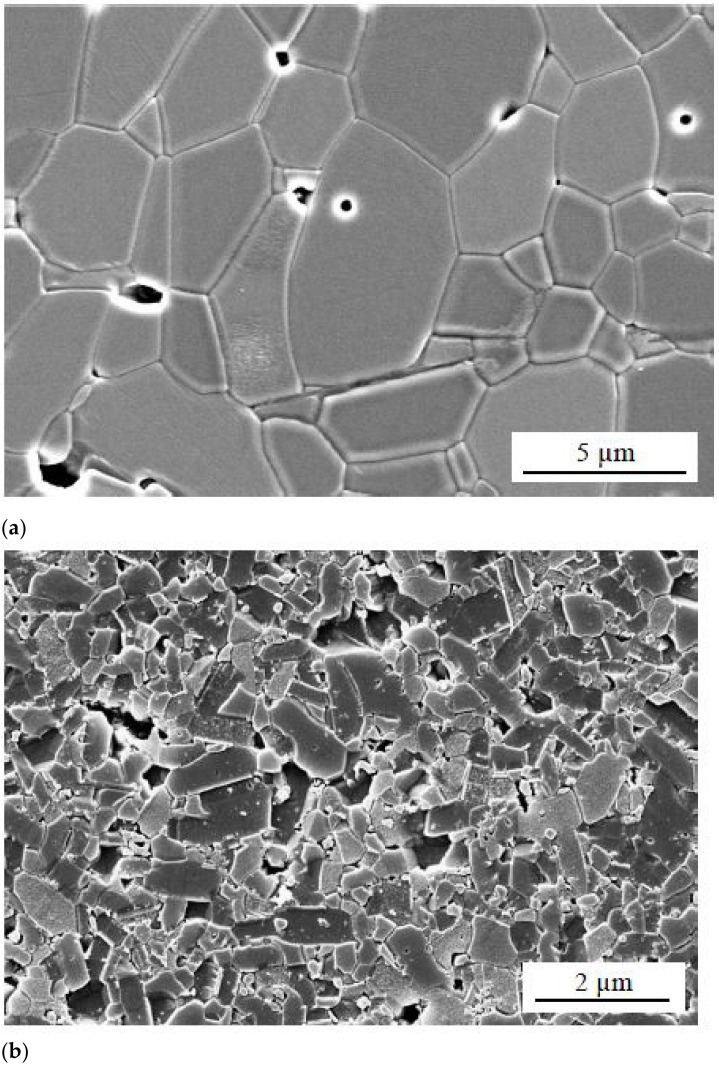
Microstructure of a polished surface of (**a**) alumina and (**b**) silicon carbide [14].

Nour et al. [14] discussed that damage due to cavitation erosion is usually found in high-speed impellers, valves, pump casings and water turbines. Generation of shock waves due to symmetric bubble implosion and formation of microjets due to asymmetric bubble implosion are two important mechanisms of cavitation wear. The microjets produce local material stress from 100 to over 1000 MPa. The surface roughness of a ceramics material has a significant impact on cavitation wear. Microstructures of polished surface of the alumina and silicon carbide are compared as shown in Figure 5. It can be seen that the alumina consists of coarse grains (~8 µm) joined by a glassy grain-boundary phase with large pores, whereas silicon carbide is composed of fine grains (~2 µm) and reveals a smaller pore size. Hence surface morphology greatly effects the cavitation wear.

In addition, they also compared oils and water as cavitating liquids while testing ceramics. The results indicated that oils are a less erosive medium when compared with water because of higher water vapor pressure, lower viscosity and higher density. Wang et al. [15] proposed two different kinds of 2-phase cavitation flow computational models, i.e., the ANSYS-CFX default model and the optimum computational model. Comparisons are made between the CFD results and the experiments. The head loss curves and vapor volume allocations are compared with the experimental data. Finally, the blade load and the total pressure variation in the passage are studied.

It seems necessary that appropriate CFD methodology is required in emphasizing the significance of slurry erosion and cavitation as applying certain analytical methods and testing of equipment both have limitations.

## 3. Investigation and Identification for Slurry Erosion and Cavitation in Pumps

To estimate the erosion loss occurring at different industrial parts, investigation and identification of different impacting factors on the wear is essential. Pump performance drops for heavier liquids such as lime slurry are found from industrial data. Whenever slurry is transported through the pump, it is imperative to account for pump head losses and as a consequence efficiency losses that occur [16]. Head and efficiency ratios are defined as shown in Equations (1) and (2), respectively.

(1) $$\mathrm{Head}\;\mathrm{Ratio}\;\left(\mathrm{HR}\right)=\;\frac{Head\;developed\;by\;slurry\;at\;a\;given\;flow\;rate}{Head\;developed\;by\;water\;at\;same\;flow\;rate}$$

(2) $$\mathrm{Efficiency}\;\mathrm{Ratio}\;\left(\mathrm{ER}\right)=\;\frac{Efficiency\;of\;pump\;for\;slurry\;at\;a\;given\;flow\;rate}{Efficiency\;of\;pump\;by\;water\;at\;same\;flow\;rate}$$

Both parameters depend on the specific gravity (SGs), volumetric concentration (Cv), particle diameter of the particles (d50) and impeller diameter (Di). The value of the efficiency ratio is usually smaller than the head ratio. The reason for this is that the efficiency ratio includes the effects of both head losses as well as power losses [9,10].

### 3.1. Experimental Investigation

**Figure 6:**
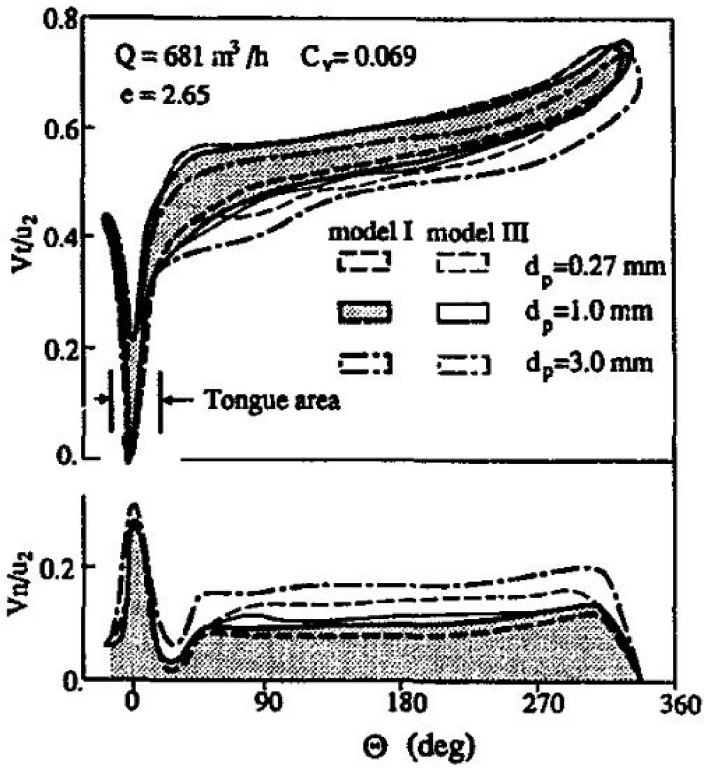
Impact velocity distribution as a function of angular position [17].

Various experimental studies have been conducted to evaluate and asses the slurry erosion phenomena. Zhong et al. [17] worked on a radial-flow pump casing to establish the effect of particle diameter and flow rate on erosion loss. As the particle diameter is increased, the erosion rate is enhanced; for diameters less than 1.0 mm the effect of particle diameter on the wear rate is negligible. They found that for particle diameters, dp = 0.27 mm and 1.0 mm, the values of impact velocity V overlap with each other. However, for dp = 3.0 mm, V increases greatly over the entire range except near the tongue (0 < 45°). It shows that the inertia of the particle increases in proportion to the cube of dp. This is shown in Figure 6.

**Figure 7:**
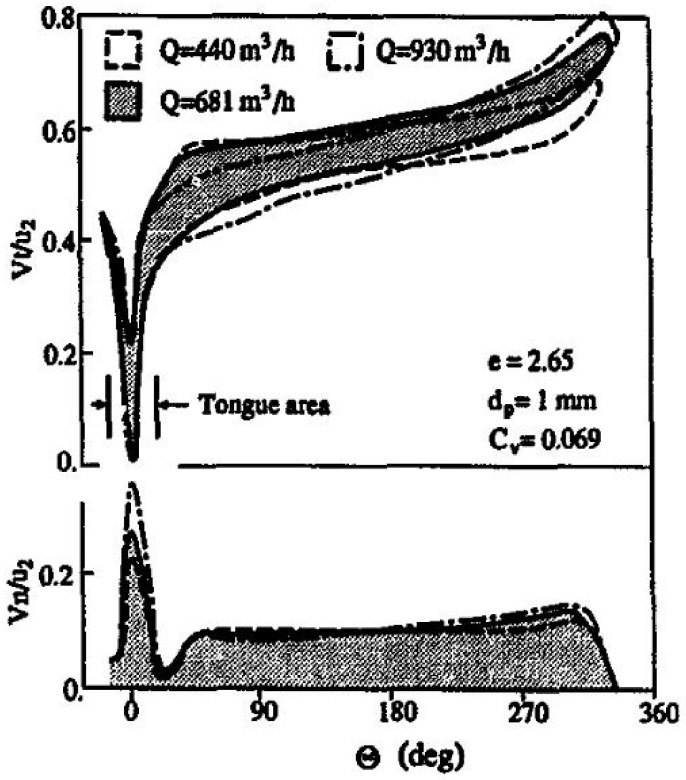
Effect of volume flow on erosion rate [17].

With an increase in flow rate, the erosion in the neighborhood of the tongue and the casing outlet gradually increases. For when dp = 1.0 mm, wear rate increases as flow rate, Q, is increased, as shown in Figure 7. The effect on wear rate near the tongue is due to a higher value of Vn and the near exit is due to Vt.

Krishnan et al. [18] determined that particle mass loading and wall shear stress both increases from the upstream of the tongue region to the downstream of the belly region. The influencing parameters, such as casing width, pump discharge, tongue curvature, particles concentration, are given importance. Tian et al. [19] studied the synergistic effects between corrosion and erosion through Coriolis sliding erosion testing setup. The larger particles produced a higher mass loss rate. However, smaller particles, such as 10 μm, have more influence than large particles at reasonably high corrosion values in removing the corrosion products from the parent material. Temperature rise considerably increases the corrosion severity. Only 15.5 °C temperature change increased the mass loss rate by 19.6–126.3% at various particle sizes. Walker and Bodkin [20] established empirical wear relationships for pump side-liner by conducting different tests. They found that wear rate is independent of varying solids concentration and impeller tip speed. It was concluded that these relationships for slurry pumps are significant for plant operators. Furthermore, they compared the erosion wear life between the field applications with a similar material under laboratory conditions. Table 1 shows the correlations developed by various researchers for slurry erosion. Wang and Qian [21] analyzed the performance of double-suction CPs which are widely used in irrigation systems. The results showed that the values of head and the shaft power are higher as the sediment particle concentration and size are increased as compared with that of pure water. Furthermore, they have developed a relationship which depends on sediment characteristics for the head reduction aspects.

**Figure 8:**
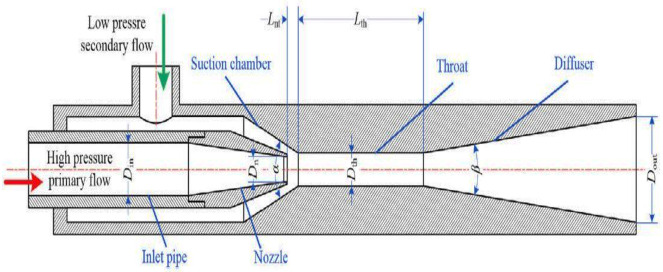
Schematic drawing of a JPCR [28].

Adamkowski et al. [26] investigated the reasons of the shaft fractures of pumps installed for the cooling system of generator sets. Spectral analysis is performed for pressure fluctuations produced by the pump blade. They studied the resonance as a result of cavitation which causes almost 20% of the impeller mass loss. Moreover, for a pump operating for 3000 h, the maximum loss in weight goes up to 23% of the initial weight of the impeller. Blau et al. [27] analyzed the repeatability and reproducibility of wear data that result from a variety of sources, including material homogeneity, choice of units of measure and choice of experimental variables. They studied different forms of wear such as cavitation erosion, three-body abrasion, solid particle erosion, dry sliding wear and fuel lubricity using the ball-on- cylinder (BOCLE) test. Long et al. [28] analyzed a jet pump for potential applications. Experiments were conducted on three diverse area ratios under varying operating conditions for its practical applications. The structure of a jet pump cavitation reactor (JPCR) mainly consists of five parts, namely, intake pipe, jet nozzle, suction space, throat and diffuser, as shown in Figure 8.

**Figure 9:**
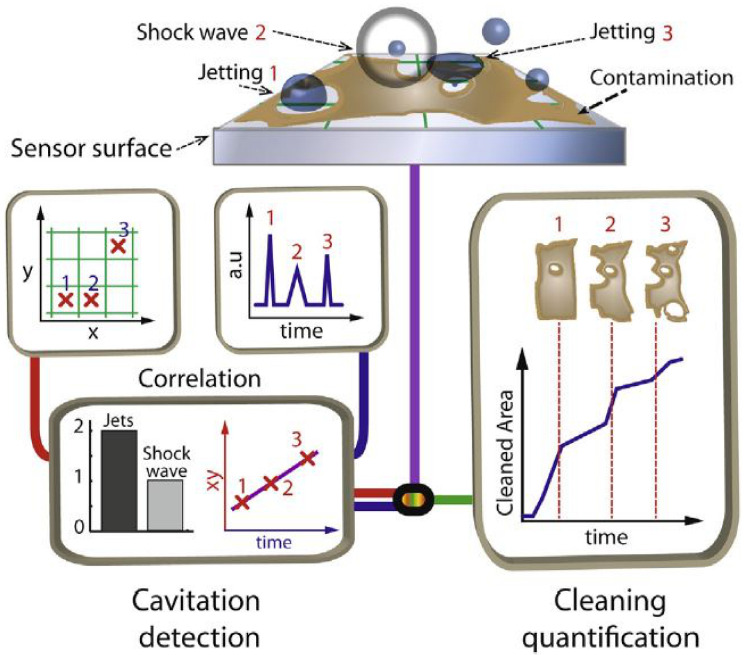
Identification of cavitation and amount of cleaning through ideal sensor [32].

They discussed the rotor dynamic fluid forces acting on a centrifugal impeller. At a positive whirl ratio, cavitation always destabilizes while it can stabilize at negative whirl ratio. Hutli et al. [29] studied the influence of hydrodynamic conditions, such as outlet jet velocity, nozzle shape and cavitation number on the behavior of the cavitating jet, where the cavitation of commercial copper is used as an indication of the cavitation behavior. However, they observed that erosion becomes more visible with decreasing cavitation numbers, as well as with increasing outlet jet velocities. Gu et al. [30] compared the cavitation pressure between the ultrapure water and water containing SiO_2_ nanoparticles. Both water samples having distinct particle loading and sizes are utilized. Furthermore, they concluded that water can be stabilized with SiO_2_ nanoparticles. Chan [31] presented a technique for cavitation identification. It is analyzed that cavitation can only occur when vapor bubbles burst near the concerned boundary. It is also discussed that below the critical NPSH the cavitation rate fluctuates against NPSH. Verhaagen and Rivas [32] have discussed the benefits and limitations of techniques for measuring the presence and cavitation magnitude. They have presented the idea of ‘‘ideal sensor’’ which can show the bubble generation while calculating the removal of impurities at the relevant temporal and spatial scales. Figure 9 shows the ideal sensor. The amount of cavitation in terms of strength and extent can be utilized to classify whether it occurred due to a jet or a shockwave. Cleaning quantification can be directly linked to the cavitation events at the lower-right side of the detection facility.

The review discussed in this sub-section [15,16,17,18,19,20,21,22,23,24,25,26,27,28,29,30,31,32,33] concludes that experimental work completed so far for slurry and cavitation erosion is encouraging but lacks similarity with the field studies. There is a need for the appropriate design, development and execution of experiments to reasonably predict the wear in both cases.

### 3.2. Numerical Investigation

CFD has emerged as a useful tool in simulating the slurry flow as the liquid passes through different components of a pump. CFD analysts should be careful in applying the required form of mass and momentum conservation equations, use suitable discretization methods, utilize appropriate boundary conditions, apply suitable solver and the most important is to deduce and present the required results in a befitting manner. For pump wear problems, the selection of suitable erosion and cavitation model is vital. The work completed by various researchers in this field is not very old but is mature enough now. By using suitable scale down models and use of symmetry where appropriate, computational resources can be saved.

**Figure 10:**
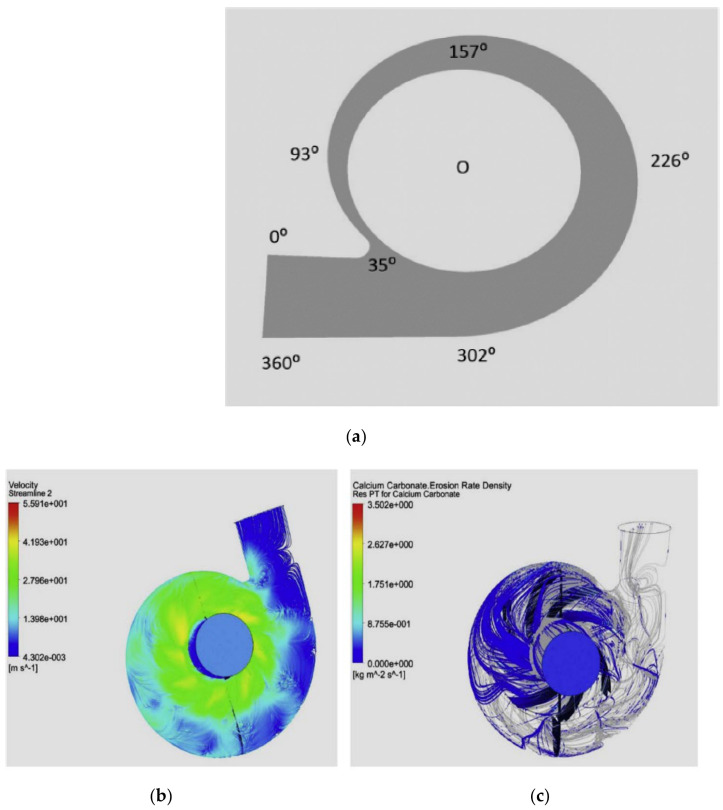

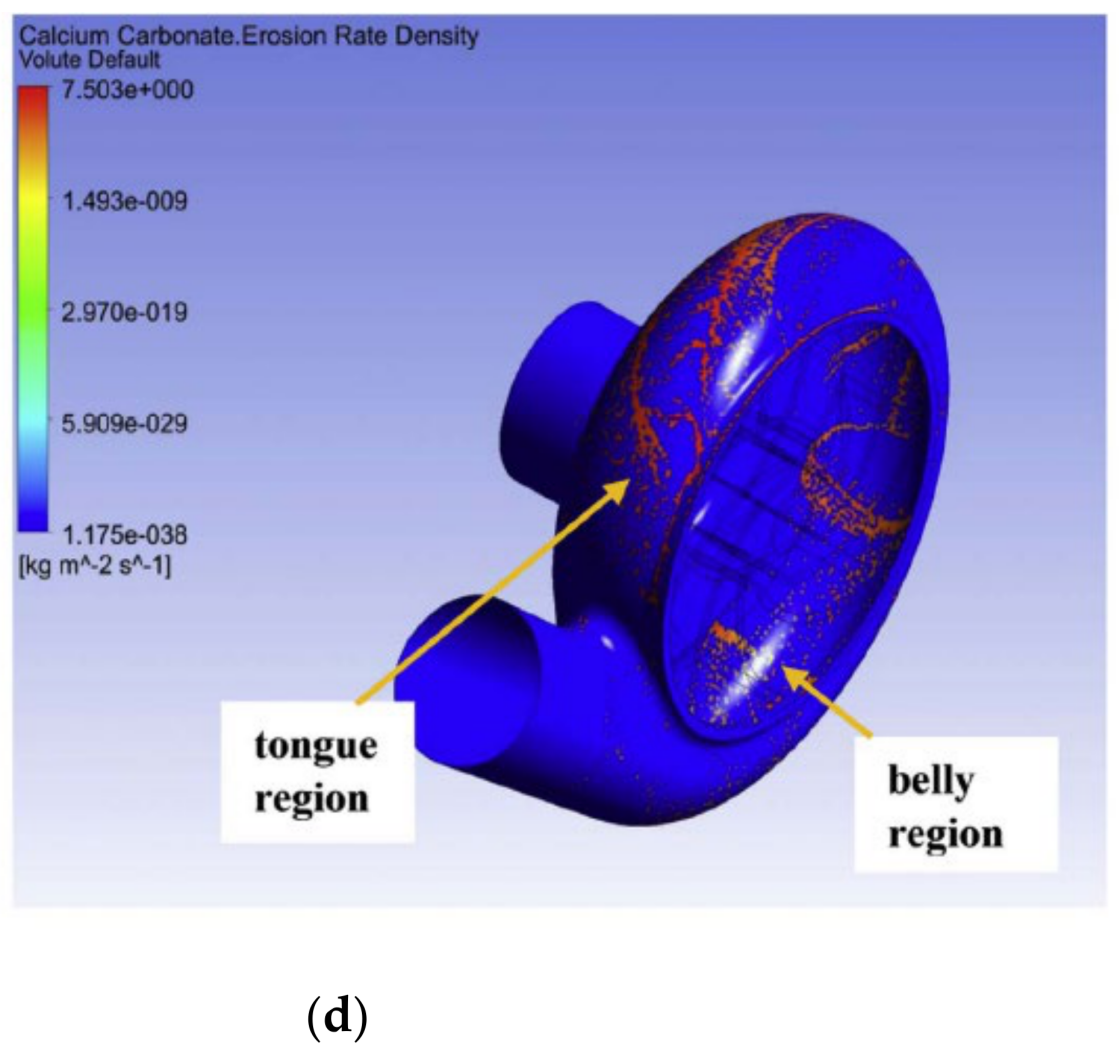
(**a**) Wear map generated for erosion identification (**b**) velocity streamlines, (**c**) particles tracking and (**d**) erosion loss [16].

Roco and Addie [33] discussed an energy methodology to estimate the erosion from the solids concentration and velocity distributions near the walls. Simulations are performed for finite volume and finite element methods for the two-phase flow in pipelines and pump components. Noon et al. [16] conducted the simulation studies for the erosion prediction and as a consequence the head and efficiency losses for lime slurry flow through CP. A wear map, along with the simulation results, are obtained as shown in Figure 10. Figure 10a shows the wear map for erosion identification in the clockwise direction, which starts near the tongue. It can be observed that the tongue area near (θ = 35°) and the belly region around (θ = 302°) are the most-affected locations as shown in Figure 10d. It is found that erosion loss grows with an increase in solid particles’ impingement velocity, volumetric concentration and diameter.

Graham et al. [34] provided experimental results using a coordinate measuring machine. A cylinder is placed in cross flow inside a pipe spreading from the pipe wall to its centerline. They quantified the pipe elbow erosion with a complicated geometry. Upadhayay [35] studied the effect of change in rotational speed, propeller geometry, original concentration and change in baffles design on the quality of suspension. They experimentally investigated utilizing the solid concentrations at several cylindrical heights and then validated them with simulation results obtained using a Eulerian–granular model.

**Figure 11:**
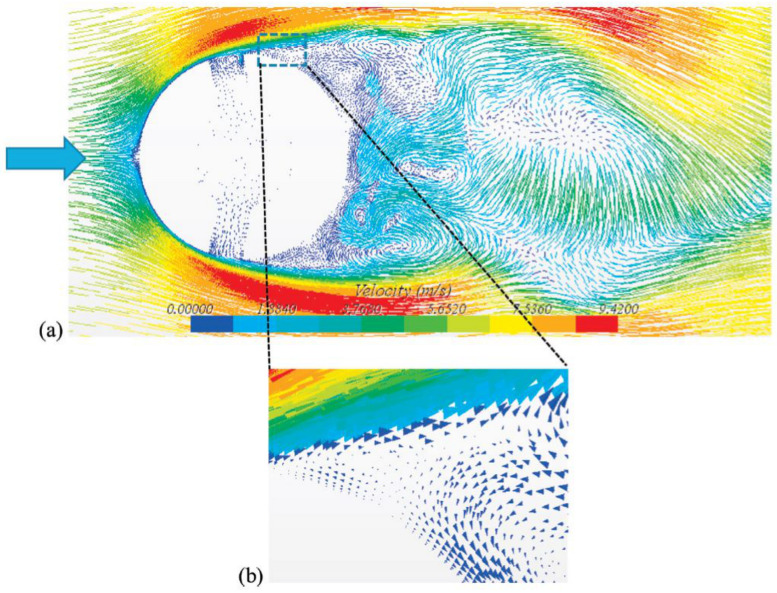
(**a**) Rapid velocity values near the pillar at Re = 2060, t = 0.07 s, (**b**) zoomed view of secondary flow zone on pillar [41].

Cavitation problem solving through numerical methods require substantial effort as the nature of the flow is quite complex. It needs fine integration and coupling between the conservation and cavitation modeling equations. Azizi et al. [36] presented a system for detection of cavitation severity in centrifugal pumps and for the improvement in its accuracy using a hybrid feature selection technique. A generalized regression neural network (GRNN) is used for cavitation identification. Xu et al. [37] studied the effects of surface topography on cavitation erosion. Regular oblique grooves, which were triangular or trapezoidal in appearance, were compared with sub-millimeter scale. Hattori et al. [38] proposed a prediction equation for cavitation wear. Material and liquid parameters were used to minimize the scattering of the prediction data. The kind of liquids, high liquid temperature, microstructure of constituents and the working life all influenced the cavitation. Liu et al. [39] performed numerical simulations with three different values of the flow coefficient, by utilizing modified k-ε turbulence model. The cavitating flow in the centrifugal pump obtained by the modified k-ε model at the design flow coefficient of 0.102 was selected. A lower value of pressure coefficient mainly occurs upstream of the impeller passage, which decreases the cavitation number, the cavity is generated on the suction side of the blade near the leading edge and then expands to the outlet of the impeller, while the downstream remains almost unaffected by the cavitation growth. The results showed good agreement with the experimental data. Fu et al. [40] studied the flow characteristics of centrifugal pumps under both steady and transient cavitation conditions. They found that centrifugal pumps used in nuclear reactors nearly become blocked and fractured due to bubble formation and burst. As a result, the axial force acting on the impeller rises and falls. It is also highlighted that transient cavitation conditions have a strong influence on the flow past in the impeller path. Nayebzadeh et al. [41] conducted experiments to study hydrodynamic cavitation in a micro channel containing a pillar. A high speed camera is utilized to capture the flow behavior. Figure 11 shows instantaneous velocity vectors near to the pillar and also indicates the flow behavior around the pillar. At the downstream of pillar, the vortex shedding is visible. The zoomed view presents the unsteady separation point at mid pillar.

**Figure 12:**
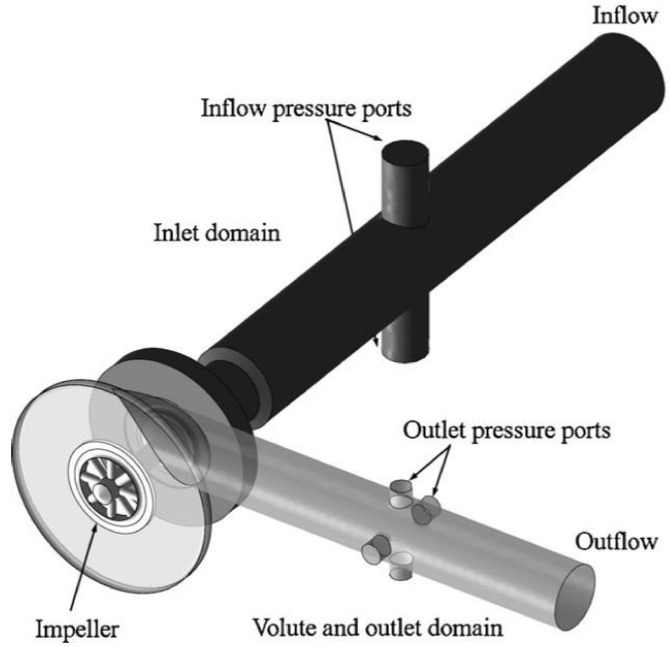
Computational domains for viscous pumps [46].

Zhang and Chen [42] validated the numerical results with the experimental hydraulic performance curves. They showed that the filter-based model is better than the standard k-ε model to predict the parameters of hydraulic performance. A Boundary Vorticity Flux (BVF) method is presented to identify the cavitating flow fields. Results indicate that the effect of cavitation exists near the blade suction surface. Shao et al. [43] studied the viscous effects on the external performance and internal flow of the pump by using five types of molten salts as working fluids through CFD analysis. They used particle image velocimetry (PIV) to measure the flow fields inside the molten salt pump and the external performance was tested by utilizing water as a working fluid. The flow velocity and performance curves have been compared with the PIV observations. Zughbi et al. [44] presented a numerical-based energy model to predict erosion rate through local flow parameters. Important empirical coefficients for the CFD model were selected and compared with the experimental data set. Zhang et al. [45] conducted both the experimental and CFD work to study the erosion phenomenon in high pressure pipelines (HPP) during fracturing slurry flow. They have studied the failure analysis of high-pressure elbows, material erosion and erosion model establishment by making use of numerical predictions. Rossetti et al. [46] performed 3D transient CFD simulations and validated the results through experimental data for first stage rotary shaft pump and second stage centrifugal viscous pump. Numerical results helped to study the flow field inside the pumps in order to compute the hydraulic efficiency of the two stages and also to show the distribution of losses inside the pumps. Figure 12 shows the computational domains developed for the analysis.

**Figure 13:**
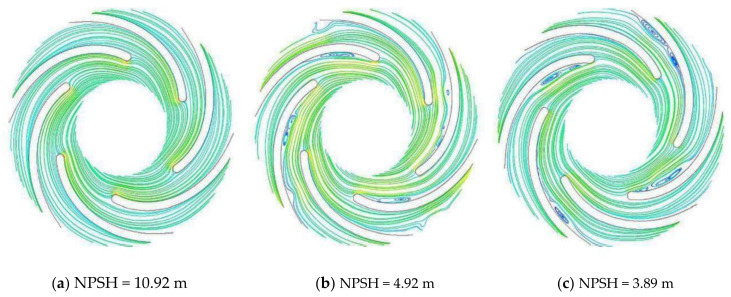
Streamline behavior for impeller under different NPSH (**a**–**c**) [47].

Ye et al. [47] investigated the cavitation phenomenon in a CP through flow visualization experiments at various flow rates. Experimental and numerical results are compared for the pump head, cavity lengths and vapor fraction. They studied the relationship between the semi-analytical model with the experiments in the broad range of NPSH in comparison to Zwart model. Figure 13 shows the streamlines for three different NPSH at 60% of design flowrate, Qd inside the impeller. Both the cavity and the vortex around it develops as NPSH decreases. It is found that cavitation is an imperative source of vortex generation.

Transient phase CFD simulations were also executed to obtain a better understanding of the flow field and to associate the separation angle to the attached cavitation angle. Brunhart et al. [48] devised a methodology to evaluate the viability of several cavitation erosion risk indicators. The distribution and intensity of the resulting ERIs were evaluated. It was anticipated that using these risk indicators will be useful product design and development which will save considerable time and cost.

Ramirez et al. [49] used a design of experiment technique for the characterization and optimization phenomena in dredging centrifugal pumps. Various parameters such as swing speed, dredging depth and inclination and impeller rpm were analyzed.

The use of simulation packages such as CFX, FLUENT, COMSOL, etc. have made life easy in slurry erosion problem solving but still a lot of effort is required to obtain accurate and precise results with minimum computational efforts and resources.

## 4. Erosion and Cavitation Reduction Techniques

Many investigators have made efforts to reduce erosion and cavitation in pumping system through different ways. However, a very scarce amount of work is found in which the economic aspect has been discussed in detail to reduce these undesirable phenomena. Comprehensive analysis for wear reduction has been discussed as a function of flow modifications, design optimization, coatings and change in target surface materials in the following sections.

### 4.1. Flow Modifications

**Figure 14:**
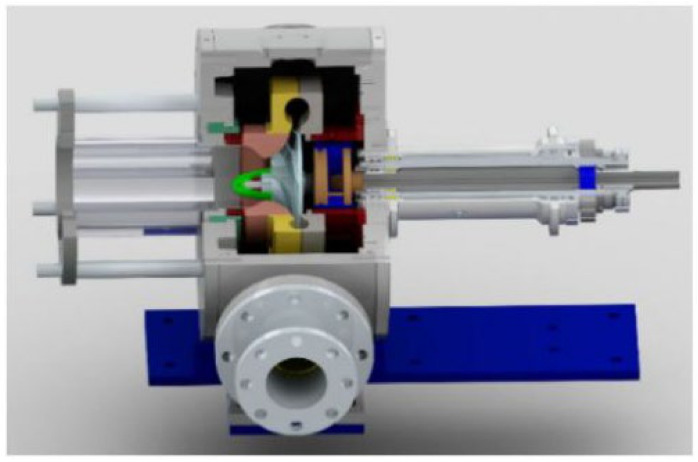
Cut-section for the rotor dynamic test facility [55].

Various techniques are adopted for erosion and cavitation reduction, but swirl motion generation at the bend and curved sections is a very useful way highlighted by numerous investigators. Wood et al. [50] studied that in order to reduce erosion at critical locations in slurry pipelines, one needs to apply swirl motion at the pipe bends. They applied parallel visualization techniques to particle-laden liquids to study the flow patterns. Lower flow rate conditions, particle impingement and particle distributions resulting from such swirl flow are analyzed in terms of latest erosion models and the likely reduction in erosion rates. Enayat et al. [51] studied that the strength of the secondary flow is influenced by the initial inlet flow conditions and the boundary layer growth. However, the secondary flow develops initially near the inside bend and decays afterwards. Furthermore, they found that curved fluid-wall interfaces prevent the calculation of cross-stream velocity components. Tarodiya et al. [52] reviewed the important measured and simulation studies on the wear estimation and performance of centrifugal slurry pumps. Different correlations were analyzed by various researchers to estimate the pump performance regarding handling slurry. Different techniques were used to identify the zones of maximum localized wear and ways to minimize it. Messaadi et al. [53] performed their work both experimentally and numerically. It has been shown that the interacting angles and quantity of friction played a pertinent role in surface failure. Surface shear effects are found at low impact angles and plastic deformation is found at high impact angles i. Graham et al. [54] investigated that a change in the fluid motion design may reduce the vortex action and thus reduced erosion. They conducted tests on small scale models which can be used to quantify the expected improvements in erosion. Valentini et al. [55] conducted an experimental classification of the rotor dynamic fluid forces acting on a whirling centrifugal impeller at distinct flow rates and cavitation conditions. Cut section in Figure 14 shows the arrangement of this facility.

### 4.2. Design Optimization Studies

**Figure 15:**
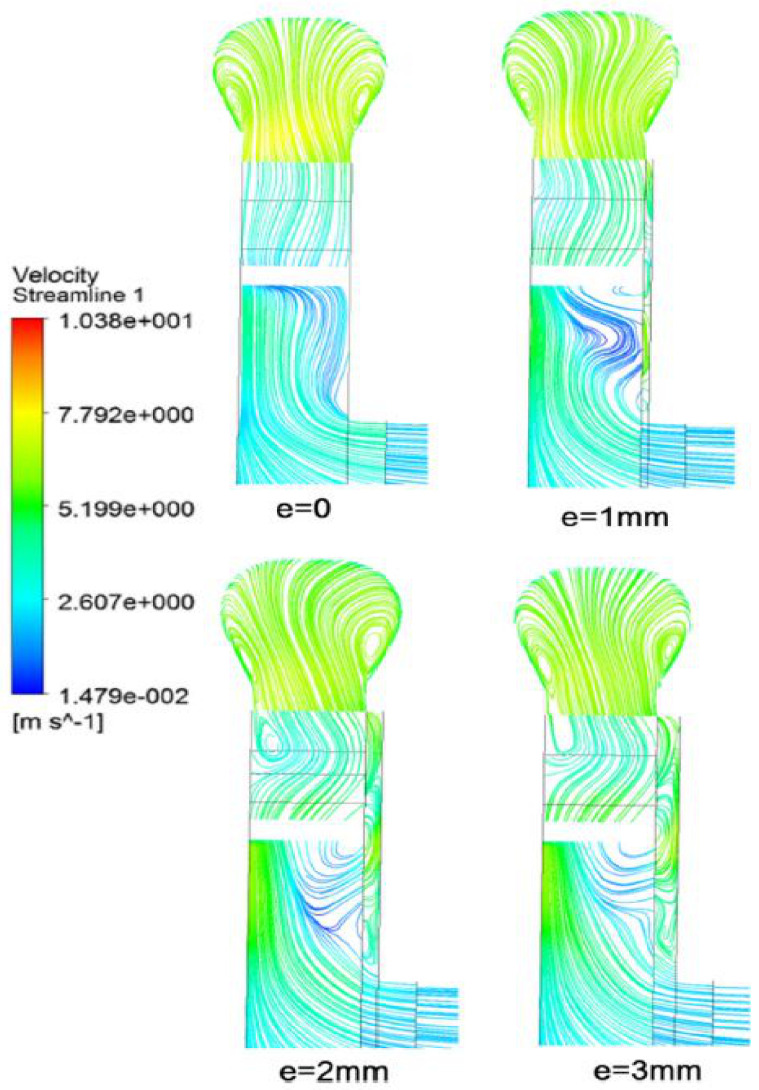
Velocity streamlines at a semi-open impeller [60].

Pump performance enhancement depends upon its efficient working; for this purpose, the erosion and cavitation reduction are indispensable. Many researchers have focused on improving pump efficiency by optimizing shape of impeller blades, clearance between impeller and casing, etc. Later on, the effort has been made to develop algorithms to optimize the pump geometry and flow conditions. Currently, metamodel multi-objective optimization techniques and machine learning tools based on neural networks technology are gaining importance and significance increase in the pump performance has been achieved by many investigators. Jie et al. [56] showed that increasing the curvature radius will change the flow field inside the pipe and as a consequence the maximum erosion region can be identified. Shi et al. [57] investigated the effect of few geometrical characteristics on the pressure distribution in the pump by utilizing a broad parametric study. The important parameters considered are sidewall clearance, the cutwater gap, vane arrangement and snubber gap. They suggested that the minimum cutwater gap should be 6% of the impeller diameter, vane arrangement should use a 30⁰ stagger, diametric snubber gap should be approximately 0.64% of the impeller diameter and the sidewall clearance should be 100%. Olszewski [58] studied the technique for optimizing an intricate pumping system with set of parallel CPs. In total, three prediction strategies are suggested: flow rate balancing, reduction in power consumption and enhancement in overall efficiency. Spence and Teixeira [59] investigated different geometric parameters, namely the vane arrangement, snubber gap, cutwater gap and the sidewall clearance. The cutwater gap and vane arrangement have the greatest impact on different locations and the flow range. Ayad et al. [60] performed a parametric study based to examine the effect of the side clearance width on the semi-open impeller to enhance the pump performance. The influence of secondary flow on the main flow is enhanced through increasing impeller side clearance width which generates vortex establishment at the blade tip as shown in Figure 15.

**Figure 16:**
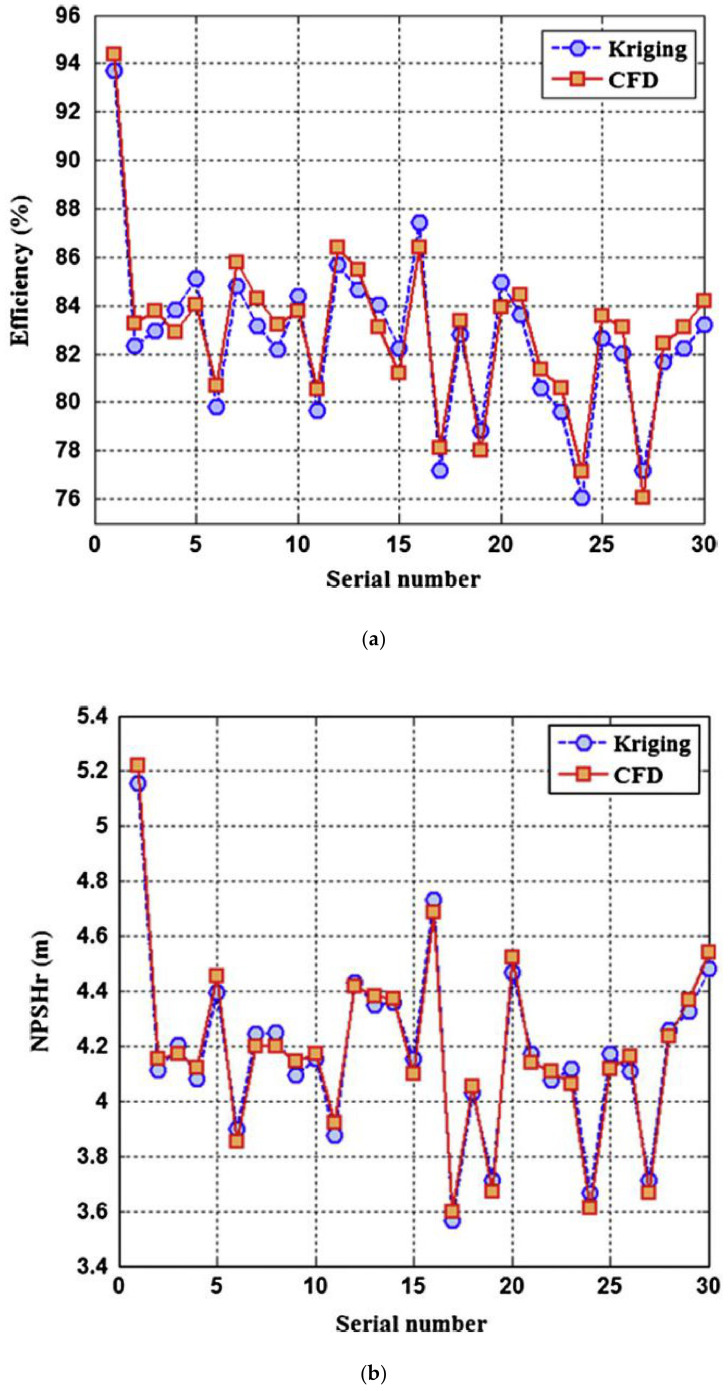
SKE approach and CFD comparison (**a**) efficiency, (**b**) NPSH_r_ [65].

Ramasamy and Ganesan [61] investigated the performance through design optimization of the pump impeller. Impeller blade design, blade thickness and angle, manufacturing techniques and required power consumption are studied. Kim et al. [62] performed multi-objective optimization to simultaneously maximize the efficiency and pressure rise for a centrifugal fan having splitter blades by utilizing 3-D RANS equations and evolutionary algorithm. Splitter location and the impeller height ratio between inlet and outlet were selected for the optimization. Heo et al. [63] conducted an optimization study for a CP containing backward-curved blades with a specific speed of 150. Efficiency and total pressure are taken as the objective functions and they used various surrogate models coupled with three dimensional Reynolds average Naiver–Stokes analysis to examine the performance characteristics of the CP. Zhang et al. [64] proposed a novel multi-objective optimization method using an SKE approach for a family of double suction centrifugal pumps with different blade profiles. The required net positive suction head (NPSHr) and hydraulic efficiency are chosen as the conflicting design variables. Figure 16a,b shows the comparison of the prediction values and the CFD simulations.

Wang et al. [66] conducted a study for design optimization for a characteristic multistage CP based on energy loss model and Computational Fluid Dynamics (ELM/CFD). They have reduced the volumetric and inter-stage leakage losses to increase the efficiency of CPs. Table 2 shows a summary of latest and important techniques utilized for increased efficiency and reduced power consumption.

### 4.3. Target Surface Material

**Figure 17:**
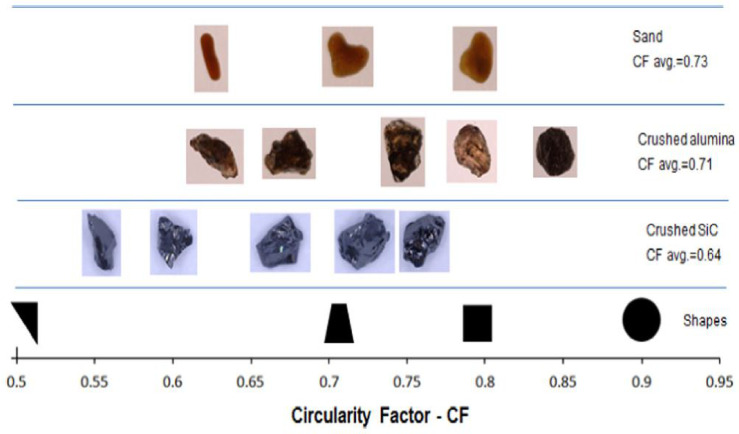
Representation of particle shapes before testing [70].

Nature of the target surface material is very important. Material properties such as hardness, toughness, resilience, etc. and mechanical properties such as tensile stress, ductile or brittle behavior play a significant role in selecting the erosion and cavitation models. Javaheri et al. [69] provided a comprehensive review relating to the slurry erosion of steels for pipeline applications. They discussed the mechanisms involved, various types of erosion test rigs and the parameters influencing the erosion rate in various steel microstructures. Combined effects of erosion and corrosion are also discussed. Walker et al. [70] did work on the study of mineral processing equipment. They found that slurry particle shape is important variable in determining erosion, especially in the grinding circuit. They used geometric parameters for shape, such as Circularity Factor (CF) and spike parameter (SPQ). For a wide range of particle shapes, a linear relationship between CF and SPQ is observed to exist. Images of the distinct particle’s shape and their circularity factors are shown in Figure 17.

They concluded that, for ductile steels and hard white irons, erosion rate decreases with higher values of particle CF, and an inverse power law relationship exists for wear rate of white irons with CF, but specific wear rates will depend on particle size, density, and impact angle, as the hardness of silica, alumina and hard SiC are higher than mineral ores. It is difficult to test these for wear in the mineral processing industry; the power law relationship calculated the relative impact for 27% Cr white iron variation in CF on erosion rate. Krella [71] analyzed the mean depth and material loss of the AlMg2 alloy and the existence of hard intermetallic nature accelerates erosion. It also initiated the development of dislocation structure and effected the hardness of AlMg2 alloy. Xie et al. [22] analyzed the erosion modes and its resistance for commonly used materials in slurry transport. Different slurry tests are used to simulate these erosion modes and to illustrate the wear resistance of the materials. Wear resistance of different materials with their mechanical properties are also discussed. Sun et al. [72] analyzed the stress condition under tensile loading for steel and iron material for erosion rates. The ploughing action is important around 30° impact angle and indentation mechanism near 90° for erosive wear mechanisms regarding the erosion resistance of the CrMo steel. Gautam et al. [73] discussed the micro cutting and ploughing for brass alloy under different operating conditions through SEM images for erosion mechanism. Slurry erosion loss was enhanced with increase in impact angle up to 60° but after that, the increments in the impingement angle were reduced for brass alloy. Alam and Farhat [74] studied the slurry erosion characteristics of various steel materials to investigate the effect of slurry particle concentration on erosion rate and erosion mechanisms. Micro-cutting, heavy plastic deformation and brittle fracture is recognized as leading erosion mechanisms. Mesa et al. [75] analyzed the mass removal mechanisms from the specimens surface. Furthermore, three martensitic stainless steels are investigated to test the effect of slurry wear resistance and temperature on electrochemical corrosion.

### 4.4. Other Interesting Techniques

The techniques discussed in this section are in their preliminary phase and will take time to become mature. Tang et al. [23] studied the interesting behavior; that by increasing the fluid viscosity, the erosion rate decreases significantly. Fluid viscosity enhances the tearing effect and limits the solid erodent’s motion and minimizes the impact energy, which results in the erosion loss reduction. Jain et al. [76] discussed various turbines appropriate for micro-hydel power plants and mainly the historical development of PAT. They reviewed the theoretical, experimental and numerical studies carried out by different researchers on PAT. They compared PAT with the conventional turbines on operation and cost basis. Selection criteria for pump running as turbine and applications of PAT in water supply pipelines are also discussed. Vikas et al. [77] discussed that smoother pipe suffers with less erosion, while rough pipes erosion was found to be 3.2 times higher. Lain et al. [78] studied that an increase in wall roughness decreases the penetration ratio significantly. They also analyzed the effect of shape changes on the erosion pattern. By increasing particle mass loading [79], the maximum penetration ratio is decreased due to the growth of inter-particle collisions and accompanying reduction in particle-wall impact velocity and angle. Aldi et al. [80] studied the performance of pump handling non-Newtonian fluid. The machine was numerically tested in order to pin point the efficiency of a low concentrated tomato paste. The improvement in impeller design made the machine suitable for the handling slurry flows. FSI, coalesced effects, surface roughness, etc. are rarely discussed by the researchers; research can be conducted to cover these important aspects in future. Gerelli et al. [81] observed that additive manufactured AlSi10Mg alloy shows outstanding cavitation erosion resistance, in comparison to the cast alloy. Very limited mass loss and erosion rate measured during the tests for AlSi10Mg alloy.

Ahmed [82] investigated the cavitation reduction through vibration techniques. Analysis have been conducted for time and frequency domains for cavitation reduction detection.

Table 3 shows a summary of latest and important techniques utilized for erosion reduction.

## 5. Impact on Process Industries

Centrifugal pumps are used extensively in process industries, irrigation systems, domestic applications, etc. However, consumption of CPs in process industry is the highest. The slurry erosion and cavitation both problems are associated with CPs which damages the plant equipment severely and needs to be reduced. The performance evaluation is necessary in order to minimize the power consumption, maintenance work and operational efforts which all together increases the production cost in a process industry. The head and hydraulic efficiency goes down as the solid concentration is increased.

### 5.1. Energy Efficiency Enhancement

**Figure 18:**
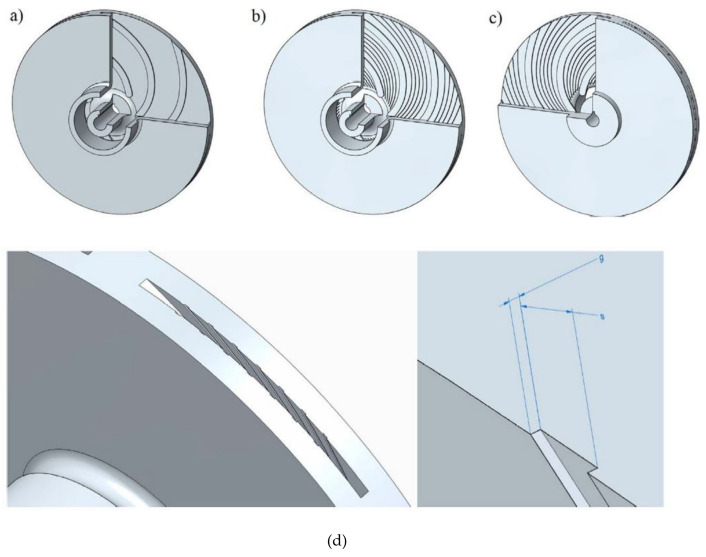
Smooth and micro-grooved impellers comparison: (**a**) smooth impeller, (**b**,**c**) micro-grooved impeller, (**d**) grooves together with characteristic dimensions [83].

In this section, the various techniques utilized by different investigators for efficiency enhancement in a centrifugal pump are reviewed extensively. Skrzypacz et al. [83] discussed the characteristics of CPs with a smooth impeller and one with micro grooves through CFD analysis. Design changes have enhanced the efficiency of CPs. The comparison of one of the grooves is presented in Figure 18.

**Figure 19:**
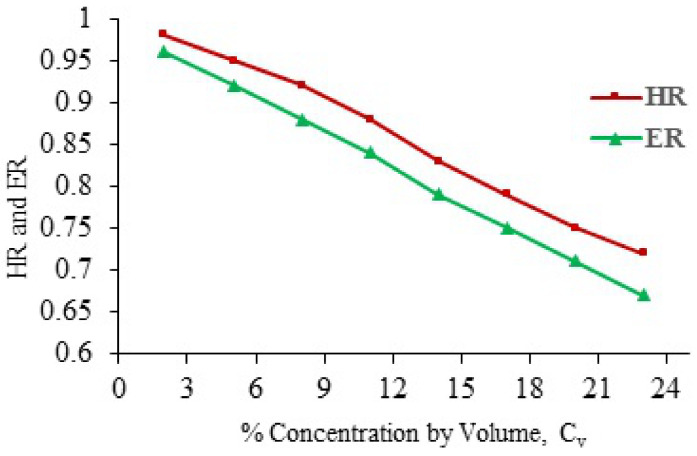
HR and ER against slurry particle concentration [16].

Binama et al. [84] presented cavitation effects which can be observed in different forms. They studied that any geometric modification can result in a totally different performance of CPs. Experimental and numerical methods are utilized to address the design process which as a result could enhance the pump system performance through erosion reduction. According to the IEA [85] 46% of the electricity produced in the world is used to run electric motors and it is about 70% of the total industrial electricity consumption. Therefore, it is necessary to enhance the efficiency of electric motors in order to reduce electricity consumption. A case study which is relevant to process industry in Pakistan is presented to emphasize the significance of energy efficiency enhancement. Noon et al. [16] presented results for the important pump performance parameters. The pump hydraulic efficiency decreases approximately to 14–15% at 18% particles concentration as shown in Figure 19.

This drop in efficiency results in huge economic losses. This trend is observed in nearly every process industry in Pakistan. Therefore, the reduction in wear and efficiency enhancement both are imperative on the basis of economic point of view.

### 5.2. Economic Analysis

CP casing is replaced on average after a 3–4 month period and adds heavy costs to the process industry. Bhat et al. [86] described that casing erosion accounts for almost 92% of repair cost in one category of CP and 72% in another. In another study, it is suggested that a near 17% reduction in energy utilization is observed through erosion reduction techniques [87].

Pump purchase decision is very important regarding economic view point. The decision can include the purchase, installation, maintenance and operation costs [88]. Industrial pumps have a variable life ranging from a minimum of 4 to a maximum of 50 years in few cases. It means that a poor decision can trouble a user for a long time. For this reason, it is crucial to study pump’s total Life-Cycle-Cost (LCC) prior to decision.

#### 5.2.1. Calculating the Total Life-Cycle-Cost

Detailed cost analyses are performed in this section for a case study discussed previously to highlight the significance of the reduction of undesirable phenomena of slurry erosion and cavitation. Nearly 30,000 centrifugal pumps are produced annually by different pump manufacturers in Pakistan [89]. Out of which, around 10,000 are used in the process industry to pump slurry and remaining are exported and for other applications. The cost comparison is made for 10,000 CPs. The average market price of a single centrifugal pump is around USD 2000. On the basis of the available data, and by making few assumptions, the pump LCC is calculated.

Table 4 and Equation (3) shows different costs important in calculating the LCC. Life cycle cost includes the following:

(3) $$LCC=\;C_{ic}+C_{in}+\;C_{o}+\;C_{e}+\;C_{m}+\;C_{do}+\;C_{en}+\;C_{d}$$

#### 5.2.2. Analyzing the Results

Detailed installation, energy and maintenance costs are evaluated [90] and a cost comparison is made for pump operation without and with slurry flow in Table 5 for three major cost components listed in Table 4. The ideal and reduced efficiencies due to wear are taken as 85% and 70% [16], respectively, for a 10-year pump life. By improving the pump design through different erosion and cavitation reduction techniques [88] as discussed in Section 4, a 3–8% efficiency increase can be enhanced.

**Figure 20:**
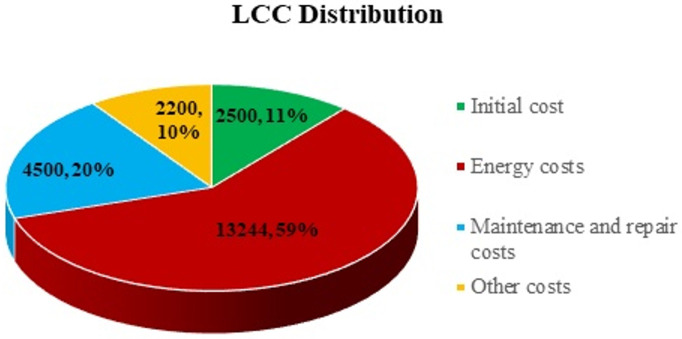
LCC distribution for pump operating without slurry.

Table 6 shows the details of all the cost components and the LCC calculations for a life of 10 years for CP usage without and with slurry flow. Figure 20 shows the LCC distribution among main cost components for flow without slurry. Energy cost is the key player, with nearly 59% of the total, whereas initial cost contribution is only 11%.

**Figure 21:**
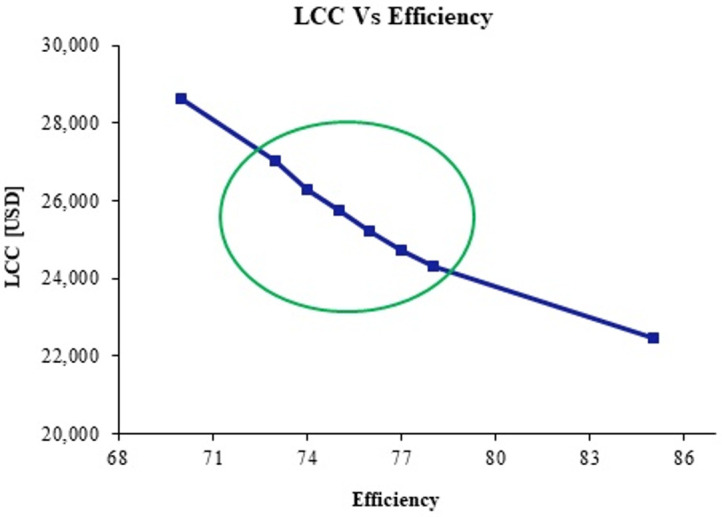
LCC against CP efficiency.

Figure 21 shows that CP LCC decreases as the efficiency is improved. The encircled points contain the efficiency values from 3 to 8% [88]. The analysis shows that a minimum of 5.9 and a maximum of 17.6% cost can be saved in the case of a 3–8% efficiency enhancement for a single CP.

On average, an 11.8% cost reduction can be achieved. Around USD 2860 can be saved for a single CP. For 10,000 CPs approximately USD 28.6 million can be saved in Pakistan alone which is a relatively small country on the basis of pump usage compared to many other countries. The erosion reduction techniques discussed can be applied to various industrial components such as pipelines, heat exchangers, valves, compressors, etc. and a lot of cost savings could be achieved.

## 6. Conclusions

The economic significance of slurry erosion and cavitation in a variety of pumps used in numerous applications have been discussed; the investigators and researchers are striving to reduce both of the wear problems. Moreover, the economic analysis executed in the current study shows a significant reduction in cost. Following are the important findings in the current review:

- Experimental work completed so far for slurry erosion and cavitation is encouraging but lacks in similarity with the field studies. Thus, there is a need for design and development of experiments to reasonably predict the wear in both cases.
- It seems necessary that appropriate CFD methodology is required in emphasizing the significance of slurry erosion and cavitation, as applying certain analytical methods and testing of equipment both have limitations.
- Slurry erosion and cavitation reduction techniques are analyzed as a function of flow modifications, design optimization, change in target surface materials and through some other novel techniques.
- Economic analysis conducted for a case study relevant to CP usage in Pakistan shows that an 8% enhancement in pump efficiency can reduce the life cycle cost to about 17.6% which could save up to USD 4281 for a single pump.

## Figures and Tables

**Table 1:** Erosion correlations developed by different investigators.

Model	Equation	Parameters
Walker et al. [6]	$\mathrm{Wear}\;\mathrm{rate}={\mathrm{KCF}}^{m}$	m = −10 K will be a function of particle concentration, density, size, etc. CF = Circularity factor
Xie et al. [22]	$M={\mathrm{At}}^{B}$	M = Mass loss and t is time Values of A and B are calculated using the least square fitting method from the test data.
Tang et al. [23]	$W=\left[\frac{\left(W_{\mathrm{total}\;}-{\;W}_{\mathrm{erosion}}\right)/W_{\mathrm{total}}}{A}\right]$	W is the weight loss rate, W_total_ is the mass of material before the test, W_erosion_ is the mass of material after erosion test and A is the affected area of polished surface.
Aponte et al. [24]	$N_{E}=\frac{E_{r}}{V_{j}A_{0}\rho_{H_{2}O}\left(\frac{C}{1-C}\right)}$	N_E_ is the dimensionless normalized erosion, E_r_ is the erosion rate in kg/s, V_j_ is the average jet velocity in m/s, A_o_ is the cross-sectional area of the jet’s outlet, C is the sand concentration and ρ_H2O_ is the water density in kg/m^3^.
H. Liu et al. [25]	$E_{r}={\left(\frac{d_{p}\rho_{g}}{\mu_{l}}\right)}^{0.5}\frac{V_{m}^{2.5}}{R.H_{v}.D}$	V_m_ is the mixture fluid velocity, which equals to the summation of superficial gas velocity (V_sg_) and superficial liquid velocity (V_sl_); R is the elbow curvature radius.

**Table 2:** Optimization methods utilized for various applications.

Ref.	Author	Objective	Optimization Methods	Algorithm Based on	Input for the Algorithm
[58]	Olszewski	minimization of power consumption	genetic algorithm(GENOCOP)	(C++)	pressure and flow rate
[61]	Ramasamy and Ganesan	reduction in power consumption	geometric and flow modifications	------	blade thickness, blade angle
[62]	Heo et al.	to increase total efficiency	Design optimization	Surrogate models	blade hub inlet angle, hub contours, blade outlet angle, and blade angle profile
[64]	Zhang et al.	increase efficiency and reduce required net positive suction head	Multi-objective optimization	Non-dominated Sorting Genetic Algorithm II (NSGA II) and Multi-Objective Evolutionary Algorithm based on Decomposition (MOEA/D)	blade angles and hub radii
[67]	Wang et al.	minimize energy losses	ELM-CFD optimization	-------	disk friction, volumetric leakage, interstage leakage, hydraulic losses
[68]	Kim et al.	efficiency and pressure rise	hybrid multi-objective evolutionary optimization	surrogate models using Latin hyperbolic curve	location of splitter, and the height ratio

**Table 3:** Important and latest techniques utilized for erosion reduction.

Study Title	Reduction Methodology	Remarks
Upstream swirl-induction for reduction of erosion damage from slurries in pipeline bends	Swirl flow resulted in lower particle impingement and particle scatter by utilizing most recent erosion models in order to reduce erosion rate	This technique is used by many investigators and mature enough now
Fluid-induced rotor dynamic forces on a whirling centrifugal pump	Positive whirl ratio has always a destabilizing effect on cavitation and leads to reduction in wear	The technique is effective and found in several studies
The influence of micro grooves on the parameters of the centrifugal pump impeller	CP impeller with micro blades/micro grooves reduced the slurry erosion and enhanced the CP efficiency	The work is novel and recent but at the preliminary stage
A novel experimental facility for measuring internal flow of Solid-liquid two-phase flow in a centrifugal pump by PIV	Geometrical changes like 6% cutwater gap and 0.64% diametral snubber of the impeller diameter, 30⁰ stagger in vane arrangement, and 100% sidewall clearance all are done	A porous inverted cone was designed to produce shear velocity difference in different swirl flow layer produce mixing effect which is a novel technique
Multi-objective optimization of double suction centrifugal pump using Kriging metamodels	Combination of simulations and experiments is used to reduce required net positive suction head (NPSHr) (which leads to cavitation reduction) and to enhance the efficiency	Latest algorithms are utilized; optimal values of design variables are identified
The effect of fluid viscosity on the erosion wear behavior of Ti (C,N)-based cermet’s	Fluid viscosity increases the solid erodent’s motion and decreases the impact energy, which results in the reduction of erosion rate	The technique is new and effective but the real time fluids used in industry are not always and much viscous
Simulation of turbulent flow through tarbela dam tunnel 3	Increased the particle mass loading to increase penetration ratio which reduces the inter-particle collisions and consequently reduces erosion reduction	It needs particle concentration optimization which is difficult to handle

**Table 4:** Important parameters in calculating the (Life-Cycle-Cost) LCC.

Symbol	Description
C_ic_	initial costs, purchase price (pump, system, pipe, auxiliary services)
C_in_	installation and commissioning cost (including training)
C_o_	operation costs (labor cost of normal system supervision)
C_e_	energy costs (predicted cost for system operation, including pump driver, controls, and any auxiliary services)
C_m_	maintenance and repair costs (routine and predicted repairs)
C_do_	downtime costs (loss of production)
C_en_	environmental costs (contamination from pumped liquid and auxiliary equipment)
C_d_	decommissioning/disposal costs (including restoration of the local environment and disposal of auxiliary services)

**Table 5:** Installation, energy and maintenance cost comparison for a single CP.

**Energy Cost (USD)**		
	**Pump Operating without Slurry**	**Pump Operating with Slurry**
Pump life (Years)	10	10
Annual usage (Hours)	3640	3640
Pump efficiency (%)	85	70
Motor efficiency (%)	75	67.5
Cost (USD)/kWh	0.0473	0.0473
Cost over lifetime (USD)	13,244	17,868
**Installation Cost (USD)**		
Foundation construction	150	150
Alignment	60	60
Piping connections	115	125
Electrical connections	80	90
Start-up	50	65
Testing	45	75
Total installation cost	500	565
**Maintenance and Repair Cost (USD)**		
Preventive maintenance	500	500
Emergency maintenance	600	700
Total maintenance cost	1100	1200

**Table 6:** LCC cost comparison.

Cost Parameter	Amount Incurred for Normal Use/USD	Amount Incurred for Slurry Usage/USD
Initial cost	2000	2000
Installation	500	565
Operation costs	1200	2400
Energy costs	13,244	17,868
Maintenance costs	1100	1200
Downtime costs	1000	1200
Environmental costs	100	100
Decommissioning/disposal costs	200	200
LCC/USD	19,344	25,533

## Data Availability

MDPI Research Data Policies.

## Nomenclature

**Abbreviations**	
A	constant
B	constant
C	sand concentration
M	mass loss
m	function of particle concentration and size
R	elbow curvature radius
t	time
W	weight loss rate
BOCLE	ball-on-cylinder
BVF	boundary vorticity flux
CE	cavitation erosion
CF	circularity factor
CP	Centrifugal pump
CSE	cavitation silt erosion
CrMo	chromium molybdenum
DSRW	dry sand rubber wheel
FSI	fluid structure interaction
GRNN	generalized regression neural network
HPP	high pressure pipelines
JPCR	Jet pump cavitation reactor
LCC	life cycle cost
NPSH	net positive suction head
PAT	pump as turbine
PIV	particle image velocimetry
RANS	Reynolds average navier-stokes
SEM	scanning electron microscope
SJE	slurry jet erosion
SKE	simulation-Kriging model-experiment
SPQ	spike parameter
USD	united states dollars
**Subscripts**	
A_a_	affected area of polished surface
A_o_	cross-sectional area of the jet’s outlet
C_v_	volumetric concentration
D_i_	impeller diameter
d_p_	particle diameter
d_50_	mean particle diameter
E_r_	Erosion rate
N_E_	Dimensionless erosion rate
NPSH_r_	required net positive suction head
SG_s_	specific gravity
AlMg_2_	Aluminum di-magnesium
SiO_2_	silicon dioxide
W_total_	weight of material before test
W_erosion_	weight of material after test
V_j_	average jet velocity
V_m_	mixture fluid velocity
V_sg_	superficial gas velocity
V_sl_	superficial liquid velocity
**Greek letters**	
ρ_H2O_	water density
θ	impact angle
